# Genomic determinants of biological age estimated by deep learning applied to retinal images

**DOI:** 10.1007/s11357-024-01481-w

**Published:** 2025-01-08

**Authors:** Yu Huang, Mohammad Ghouse Syed, Ruiye Chen, Cong Li, Xianwen Shang, Wei Wang, Xueli Zhang, Xiayin Zhang, Shulin Tang, Jing Liu, Shunming Liu, Sundar Srinivasan, Yijun Hu, Muthu Rama Krishnan Mookiah, Huan Wang, Emanuele Trucco, Honghua Yu, Colin Palmer, Zhuoting Zhu, Alexander S. F. Doney, Mingguang He

**Affiliations:** 1 Guangdong Eye Institute, Department of Ophthalmology, Guangdong Provincial People’s Hospital (Guangdong Academy of Medical Sciences), Southern Medical University, Guangzhou, 510080 China; 2 Guangdong Cardiovascular Institute, Guangdong Provincial People’s Hospital, Guangdong Academy of Medical Sciences, Southern Medical University, Guangzhou, 510080 China; 3 Division of Population Health and Genomics, University of Dundee, Ninewells Hospital and Medical School, Dundee, DD1 9SY UK; 4 VAMPIRE Project, Computer Vision and Image Processing Group, School of Science and Engineering (Computing), University of Dundee, Dundee, DD1 9SY UK; 5 Centre for Eye Research Australia, Melbourne, VIC 3002 Australia; 6 State Key Laboratory of Ophthalmology, Zhongshan Ophthalmic Center, Sun Yat-Sen University, Guangzhou, 510060 China; 7 School of Optometry, The Hong Kong Polytechnic University, Hong Kong, People’s Republic of China

**Keywords:** Retinal age, Biological age, Genome-wide association analysis, Mendelian randomization

## Abstract

With the development of deep learning (DL) techniques, there has been a successful application of this approach to determine biological age from latent information contained in retinal images. Retinal age gap (RAG) defined as the difference between chronological age and predicted retinal age has been established previously to predict the age-related disease. In this study, we performed discovery genome-wide association analysis (GWAS) on the RAG using the 31,271 UK Biobank participants and replicated our findings in 8034 GoDARTS participants. The genetic correlation between RAGs predicted from the two cohorts was 0.67 (*P* = 0.021). After meta-analysis, we found 13 RAG loci which might be related to retinal vessel density and other aging processes. The SNP-wide heritability (*h*^2^) of RAG was 0.15. Meanwhile, by performing Mendelian randomization analysis, we found that glycated hemoglobin, inflammation hemocytes, and anemia might be associated with accelerated retinal aging. Our study explored the biological implications and molecular-level mechanism of RAG, which might enable causal inference of the aging process as well as provide potential pharmaceutical intervention targets for further treatment.

**Graphical Abstract:**

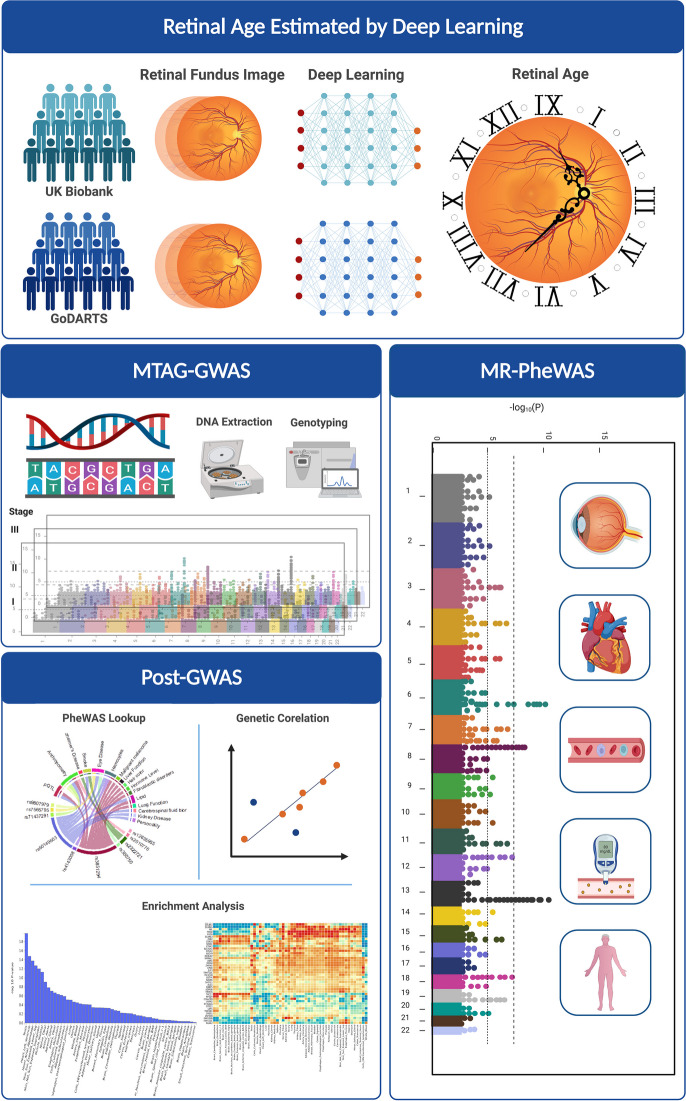

**Supplementary Information:**

The online version contains supplementary material available at 10.1007/s11357-024-01481-w.

## Introduction

Individuals vary in their susceptibility to degenerative changes associated with age [1]. Biological age (BA) or functional age may be a more important determinant of age-related health problems than chronological age (CA). Conventionally, BA can be defined by a range of hallmarks such as telomere length, epigenetic alterations, DNA methylation, or mitochondrial function [2, 3]. Estimated BA derived from such biological markers outperformed CA in predicting frailty and mortality [4] suggesting its superiority in predicting general age-related health outcomes [5].

Recently, with the development of deep learning (DL) techniques, there has been growing interest in the use of this approach to determine BA from latent information contained in medical images, for example, BA derived from brain magnetic resonance images (MRI) [6, 7], from liver and pancreas MRI [8], and chest radiographs [9]. These BAs have shown good performance in predicting overall mortality. However, according to the American Federation of Aging Research (AFAR) [10], a biomarker of aging should ideally be more efficient and accurate than CA in predicting longevity meanwhile remaining applicable to general diseases and be widely practicable and cost-effective. Thus, effective, non-specific, non-invasive, and economic aging biomarkers were still needed.

The retina comprises a highly vascularized neurological tissue and is widely regarded as a window indicating global tissue health. For example, retinal vascular features are a strong predictor for all-cause mortality and other cardiovascular or kidney diseases [11–14], while retinal neuron tissue can also be a good marker for systemic neuron-degenerative diseases [15, 16]. Importantly, highly detailed images can be conveniently acquired with relatively low-cost equipment and minimal training. This makes images of the retina highly attractive as a substrate for deep learning to investigate ageing.

Currently, several studies have reported a DL approach to predict age using retinal images. Preliminary findings suggested that the predicted retinal age gap (RAG, the difference between chronological age and predicted retinal age) was strongly predictive of mortality and a range of other conditions [17–19]. However, only one study explored its biological (genetic) causal determinants [20] but without further validation. The biological implications of RAG should be fully explored in order to enable causal inference of the aging process. Furthermore, a molecular-level mechanistic understanding of RAG might ultimately make it possible to apply pharmaceutical interventions to achieve its beneficial effects.

It is likely that the rate of tissue ageing is a consequence of both endogenous genetic factors and environmental exposures. To further improve the understanding of the RAG, we conducted a genome-wide association study (GWAS) on RAGs obtained from two distinct populations, UK Biobank, and Genetics of Diabetes Audit and Research in Tayside Scotland (GoDARTS) with different DL models. Firstly, we tested the genetic concordance of RAGs derived from different DL algorithms. Secondly, we explored the biological relevance of this trait. Thirdly, we determined biological causal factors in a Mendelian randomization (MR) framework.

## Materials and methods

### UK Biobank cohort

UK Biobank recruited more than 500,000 UK residents aged between 37 and 73 years during 2006 and 2010. Participants were invited to complete comprehensive healthcare questionnaires, provide detailed physical measurements, and provide biological samples. Other laboratory tests, medical images, or health-related events were ascertained via data linkage to hospital admission records and mortality registry. The majority of individuals who consented were also genotyped. The baseline examination resulted in the collection of 68,151 paired color retinal photographs which were used in this study. Ethical approval was obtained from the National Health Research Ethics Service (Ref 11/NW/0382) and all participants provided (digital) written informed consent. Details of the study design and data composition can be found elsewhere [21].

### GoDARTS cohort

Participants from the Genetics of Diabetes Audit and Research in Tayside Scotland (GoDARTS) were tested in the replication phase. The GoDARTS type 2 diabetes (T2D) case–control study population was recruited from the Tayside region of Scotland between the years 1997 and 2007; for each participant, the longitudinal data on biochemical investigations, prescriptions, and clinical events were assembled through data linkage to their electronic medical records from either hospital admission or primary health care. In total, more than 20,000 participants with medical records for a maximum of 30 years of follow-up were assembled. A subset of these participants had also consented for genetic analysis (genome-wide genotyping). GoDARTS has previously been described fully [22]. In this study, we used diabetes retinal screening images obtained from the Scottish Diabetes Retinal Screening Service which were all centrally collected since 2006. More details can be found at http://diabetesgenetics.dundee.ac.uk/.

The GoDARTS study has been approved by Tayside Committee on Medical Research Ethics, and informed consent was obtained from all patients (REC reference 053/04).

### Deep learning estimation of retinal age and retinal age gap

For details of the training and verification process of the DL model for age prediction in UK Biobank, please refer to Zhu et al. [18]. Briefly, within the 46,969 UK biobank participants whose retinal fundus images were available, 11,052 participants who reported no previous disease were used to train the DL model for age prediction. Of the remaining 35,917 participants, images from the right eye were prioritized for the prediction of retinal age.

In the GoDARTS study, only patients with T2D had retinal images. In total, 8570 individuals had retinal images at the baseline. During the follow-up yearly screening, a total number of 102,082 retinal images from both eyes were collected. These images were used from training to derive the retinal age, and the baseline retinal age was used for analysis in this study. Details of the DL process have been provided in Supplementary Method 1.

For both the UK Biobank and GoDARTS cohorts, we defined the difference between retinal age predicted by the DL model and chronological age as the RAG. A positive RAG indicates predicted age being older than chronological age. Firstly, the rank-based inverse normal transformation (INT) was applied to the RAG in the main study in order to normalize RAG. In the sensitivity analysis, RAG values prior to rank-based inverse normal transformation were analyzed as a continuous trait. Additionally, RAG was analyzed as a binary trait, with participants classified into a “Case group” (RAG > 3.55 years, mean absolute error) and a “Control group” (RAG < − 3.55 years) (Supplementary Method 2). To further compare the genetic effects on RAG in individuals T2D, patients diagnosed with T2D in the UK Biobank were identified (Supplementary Method 3), and GWAS analyses on RAG were performed in this cohort. The results were then compared with those from the GoDARTS study.

### Genotyping, imputation, and quality control

For the UK Biobank, genome-wide genotype data were available for 488,377 participants. For the GoDARTS participants, genome-wide genotyping was available for 25,409 patients with diabetes. Details of the genotyping and imputation process are in Supplementary Method 4.

In general, routine genetic quality control was applied before the analysis: SNPs with call rate < 95%, imputation quality score < 0.8, or with minor allele frequency (MAF) less than 1%, or those that failed Hardy–Weinberg tests (*P* > 10e − 06) were removed; SNPs on the sex chromosomes and mitochondrial were also excluded from analyses. For sample QC, individuals who do not have the retinal image, were not of European ancestry, with genotyping call rate < 95%, or showed a mismatch between phenotypic and genotypic gender, or demonstrate relatedness with others (UK Biobank: third-degree relatives or closer; GoDARTS IBD > 0.8) were removed.

### GWAS and meta-analysis

In stage 1, we performed GWAS of RAG in the UK Biobank population. We used mixed models (BOLT-LMM v2.4 [23]) and adjusted for sex, age, and the first five principal components (PC) to account for population structure. The PCs were derived from genetic data and calculated as described by Bycroft et al. [24], using genotype information from the UK Biobank population to capture ancestry-related variation and minimize confounding due to population stratification. In stage 2, for the RAG GWAS in GoDARTS, we made use of PLINK 2.0 software [25] for the GWAS analysis. However, because GoDARTS genotyping has been done on several different arrays, this was performed separately for each separate genotype dataset. To combine the results within the GoDARTS study, we meta-analyzed the GWAS summary statistics using the inverse variance weighted method (METAL software [26]).

**Fig. 1:**
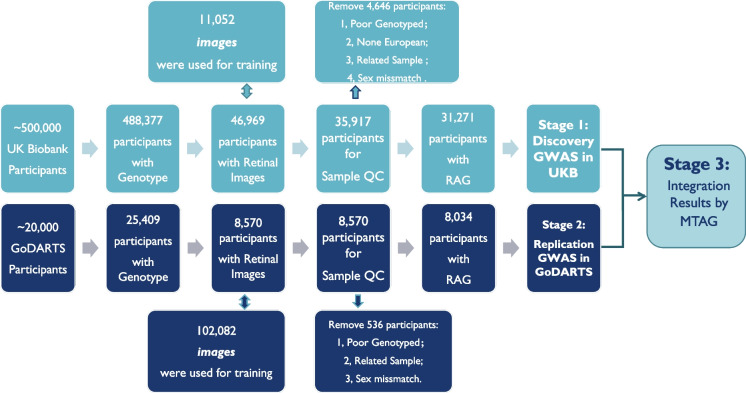
Flowchart of the study design. The three stages of this study, as well as the data resources and main quality control process for each stage

Subsequently, in stage 3, cross-validation was performed to compare the GWAS results between the two cohorts. Prior to further meta-analysis, the genetic correlations between the two GWASs were evaluated in order to test whether there was shared genetic architecture between them. Multi-trait analyses of GWAS (MTAG) [27] was run to combine the summary statistics between the UK Biobank cohort and GoDARTS cohort. By incorporating information from other genetic correlated traits, MTAG leverages the common genetic information and boosts the power of the trait of interest. Figure 1 illustrated the overall study design.

### Enrichment analysis

We conducted gene-based and pathway analysis in MAGMA, as implemented in FUMA (version 1.3.1) [28, 29]. In general, in the annotation step, a 5 kb upstream and downstream window was applied to the gene location, followed by gene-based analysis. A total of 10,894 predefined gene sets from KEGG, Reactome, BioCarta, and Gene Ontology (GO) terms were used for pathway enrichment analysis [30–33], and Bonferroni-corrected *P* values were used to define the significance threshold.

### SNP annotation and phenome-wide association analysis (PheWAS)

To identify independent genome-wide significant SNPs, we applied both clumping (*r*^2^ < 0.1 within 250 kb) as well as joint and conditional analysis (cojo-GCTA) to the summary statistics from MTAG [34]. For the significant SNPs, variant annotation was based on the Genome Reference Consortium Human Genome Build 37 derived from the University of California Santa Cruz (UCSC) Genome resource [35]. In silico functional annotation was performed by FUMA [29] which includes the annotation databases such as RegulomeDB [36] and HaploReg v1 [37]. Expression quantitative trait loci (eQTL) analysis was performed using the EyeGEx database [38] and chromatin interaction mapping was further analyzed using Hi-C [39].

To further investigate the cross-trait effects of the RAG SNPs, in PhenoScanner [40] and GWAS catalog [41], we retrieved all association results for SNPs in high LD (*r*^2^ = 0.8, European ancestry) that were genome-wide significant at *P* < 5e − 08. For further explanation and exploration, PheWAS was carried out for each individual SNP by looking up their association with other disease or lifestyle traits in the Gene Atlas catalog (http//geneatlas.roslin.ed.ac.uk).

### Comparison with other biological age genes

As further validation, genes reported from GWAS of “eye ages,” “lung age,” “abdominal age,” or “blood age” were compared with genes for RAG by Venn diagram. The tissue age genes were reported by Goallec et al. (https://www.multidimensionality-of-aging.net/).

### Genetic correlation

LD score regression implemented in LDSC [42] (https://github.com/bulik/ldsc) was used to estimate the SNP heritability (*h*^2^) of the RAG. To further investigate the extent to which the genetic variance was shared between accelerated retinal age and other relevant biological traits, genetic correlations were estimated for traits with adequate heritability (*h*^2^ > 0.1). These biological traits included ophthalmic traits, anthropometric traits, blood pressure, lipid, glycated hemoglobin, kidney function, smoking, cardiovascular diseases, Alzheimer’s disease, diabetes, and longevity. The summary statistics were downloaded through GWAS catalog links [41].

### Generation of genetic risk scores (GRSs)

Genetic risk scores (GRSs) for RAG were calculated using summary statistics derived from MTAG. Significant (*P* < 5.0 × 10^−8^) and independent SNPs (*r*^2^ < 0.1) were selected to generate weighted GRSs for each participant in the UK Biobank, utilizing the –score function in PLINK 2.0 [25]. The association of the RAG GRS with the incidence of retinal aging-related diseases (glaucoma, cataract, and age-related macular degeneration) and mortality was assessed. Additionally, the predictive power of the RAG GRS for these conditions was evaluated using the area under the receiver operating characteristic (ROC) curves.

### Mendelian randomization

Finally, to explore the potential causalities of the RAG, Mendelian randomization analysis was performed using Two-Sample MR in R package [43]. These traits include ocular features such as axial length, refractive error, intraocular pressure, glaucoma, age-related macular degeneration (AMD), and diabetic retinopathy; anthropomorphic and lifestyle traits height, BMI, smoking, alcohol consumption, and sleep duration; and biological traits blood pressure, blood lipids, blood glucose, and hemocytes. The instrumental variables for each trait were taken from SNPs reported in GWAS catalog, with certain QC processes: removing ambiguous SNPs, removing mismatched SNPs, paradomic SNPs, and SNPs in high LD (*r*^2^ > 0.1).

## Results

### Stage 1: Discovery of RAG risk loci in the UK Biobank

In stage 1, we performed GWAS in the UK Biobank dataset. A total number of 31,271 European participants were involved and 6,133,594 SNPs were tested. After Bonferroni correction and SNP clumping, 16 sentinel SNPs reached genome-wide significant level. The strongest signal was found for SNP rs60149603 (*P* = 1.4e − 95) located on chromosome 2, and 7 independent SNPs were also identified from the same chromosome. The second strongest signal was rs8001273 (5.7e − 22) located on chromosome 13 within the gene *LOC105370101*. Other SNPs are provided in Supplementary Table 1 and Supplementary Fig. 1a. The overall heritability estimated by LDSC was 13.8% and there was no evidence of inflation due to population stratification (LDSC intercept 1.01, se = 0.01).

In the sensitivity analysis, we performed linear regression on RAG values prior to rank-based inverse normal transformation and conducted logistic regression by categorizing participants with RAG > 3.55 as cases and those with RAG < − 3.55 as controls. Notably, participants with RAG > 3.55 tended to be younger and predominantly female. Detailed demographic characteristics of the cases and controls are presented in Supplementary Table 2. GWAS for the non-transformed RAG and the logistic model demonstrated similar findings (Supplementary Fig. 1b, c; Supplementary Table 3).

### Stage 2: Replication of the genetic effect in the GoDARTS cohort

**Fig. 2:**
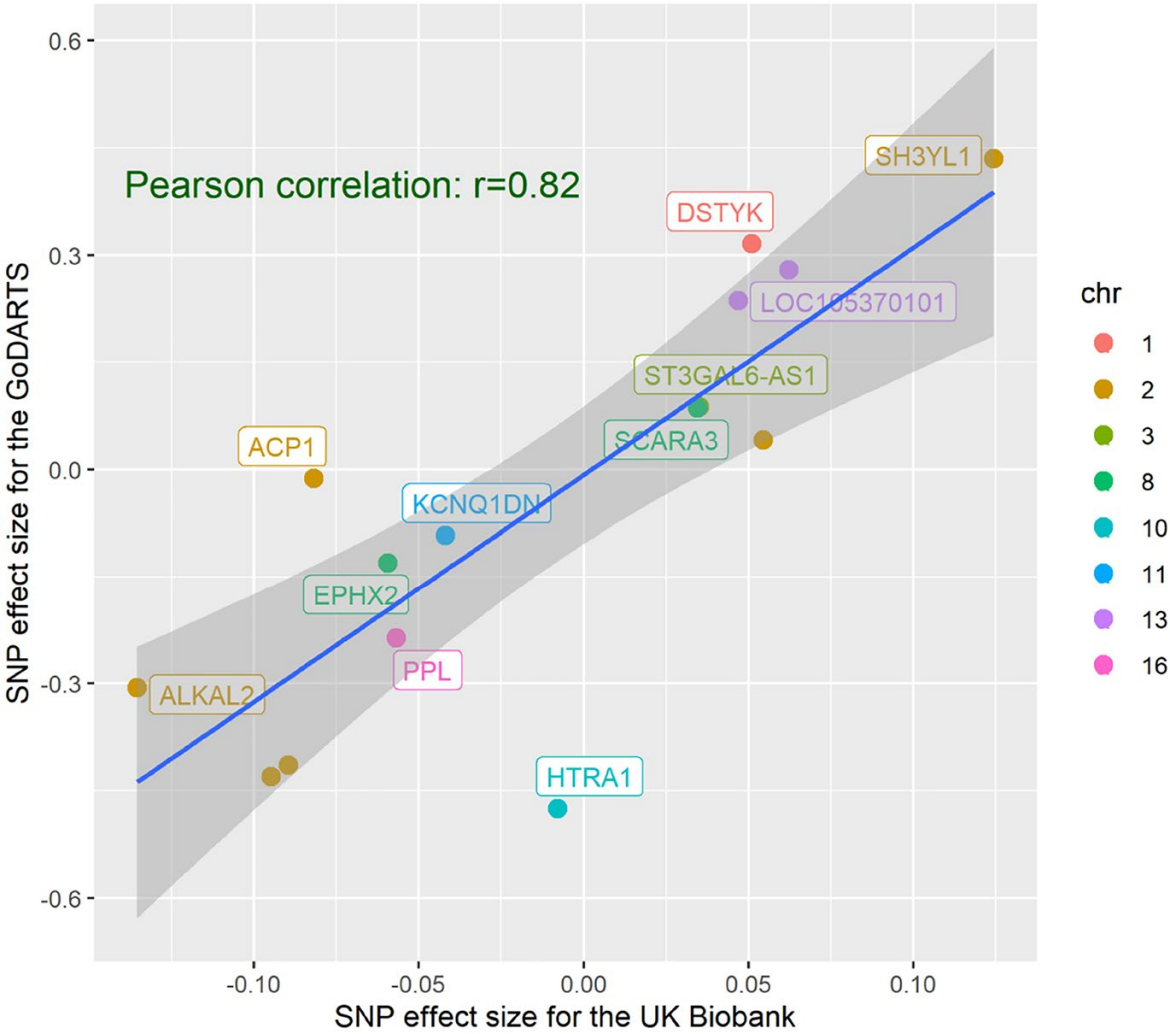
Pearson correlation of the effect sizes of the top SNPs derived from two different GWAS of RAG. The x-axes show RAG SNP effect estimates for the stage 1 GWAS using the UK Biobank population. The y-axes show the effect estimates for the same SNPs obtained from the stage 2 GWAS using the GoDARTS population. Each dot represents a SNP and the color represents SNPs from different chromosomes

Stage 2 analysis was performed in GoDARTS. After meta-analysis of different phases, 8034 participants with 8,625,586 SNPs were analyzed. Among the 16 sentinel loci in the UK Biobank study, 2 SNPs were not found in the GoDARTS study and 7 SNPs were replicated in the GoDARTS stage 2 analysis with *P* < 0.01. The effect of these SNPs was in the same direction (Supplementary Table 1). There was a relatively high concordance between the two populations for the significant loci. The Pearson correlation coefficient of the effect sizes is (*r*) = 0.82 (Fig. 2). For the overall genetic effect, we confirmed a high genetic correlation between RAG in UK Biobank and RAG in GoDARTS (rg = 0.67, *P* = 0.021). As a sensitivity analysis, we also performed a GWAS of RAG for the UK Biobank T2D population (3279 participants) and we had similar findings (Supplementary Table 1).

Apart from replicating the findings from stage 1, GWAS of RAG in the GoDARTS identified two additional SNPs that reached genome-wide significance. The first, rs932275 (*P* = 3.04e − 10) is located in the intron region of *HTRA1* on chromosome 10, a gene previously associated with age-related macular degeneration (AMD). The second, rs13274427 (*P* = 1.35e − 08), is situated on chromosome 8 within *KBTBD11/KBTBD11-OT1* region. However, neither of these SNPs was nominally significant in the UK Biobank (Supplementary Fig. 2, Supplementary Table 1). The overall heritability was slightly lower than the UK Biobank cohort with an estimated *h*^2^ of 10.1% while the inflation remained low (LDSC intercept 1.01, se = 0.007).

### *Stage 3: Discovery of novel RAG loci *via* multi-trait genome-wide association analysis (MTAG)*

**Fig. 3:**
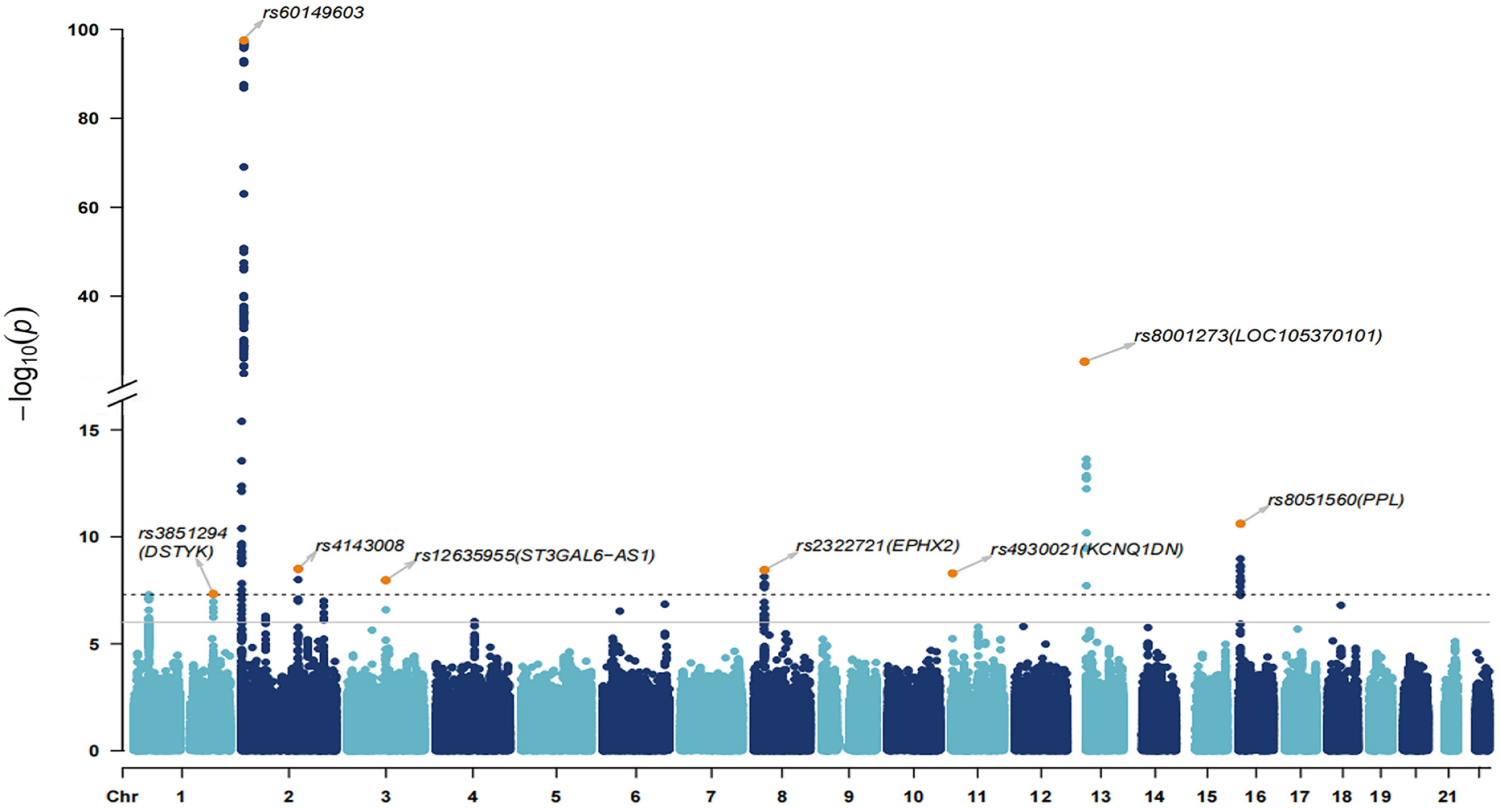
Manhattan plot displaying RAG *P* values from the multi-trait GWAS (MTAG) analysis. Each dot represents a SNP, the x-axis shows the chromosomes where each SNP is located, and the y-axis shows − log10 *P* value of the association of each SNP with RAG in the stage 3 MTAG analysis. The dash horizontal line shows the genome-wide significant threshold (*P* value = 5e − 8) and the solid gray line shows the suggestive significant threshold (*P* value = 1e − 5). The nearest gene to the most significant SNP in each locus has been labeled

The MTAG yield 13 statistically independent sentinel SNPs (Table 1, Fig. 3) from 8 leading genomic regions (Supplementary Fig. 3). This included all previously UK Biobank regions as well as 1 novel region from chromosome 1 which encodes *DSTYK* (Supplementary Table 1). Joint and conditional analyses by cojo-GCTA did not identify any further independent signals. As expected, the overall heritability was increased (*h*^2^ = 15%), and there was no sign of inflation (intercept = 1.00, se = 0.01).

**Table 1:** Retinal age gap (RAG) loci identified through stage 3 MTAG analysis

SNP	CHR	POS	A1	A2	Gene	A1_FREQ	BETA	SE	*P*	*N*
rs3851294	1	205,130,413	A	G	*DSTYK*	0.09	0.072	0.013	4.68e − 08	39,305
rs74487397	2	104,036	T	C		0.96	− 0.126	0.02	2.81e − 10	39,242
rs71437291	2	256,414	G	A	*SH3YL1*	0.95	0.155	0.017	4.24e − 19	38,278
rs60149603	2	285,421	A	G	*ALKAL2*	0.66	− 0.169	0.008	3.11e − 98	39,045
rs115961439	2	359,900	A	G		0.96	− 0.121	0.02	1.59e − 09	39,305
rs4143008	2	145,339,954	A	G		0.85	0.064	0.011	3.17e − 09	38,103
rs12635955	3	98,436,188	T	C	*ST3GAL6-AS1*	0.51	0.044	0.008	1.07e − 08	37,853
rs2322721	8	27,352,750	A	G	*EPHX2*	0.9	− 0.074	0.013	3.56e − 09	39,186
rs2010776	8	27,590,027	A	G	*SCARA3*	0.56	0.044	0.008	1.58e − 08	39,305
rs4930021	11	2,892,391	A	G	*KCNQ1DN*	0.25	− 0.051	0.009	5.15e − 09	37,863
rs8001273	13	20,693,147	C	T	*LOC105370101*	0.62	0.082	0.008	1.54e − 25	38,664
rs7324150	13	20,698,681	G	A		0.17	0.063	0.01	3.46e − 10	39,232
rs8051560	16	4,960,254	C	A	*PPL*	0.86	− 0.074	0.011	2.44e − 11	38,139

*CHR*, chromosome; *POS*, base pair position; *A1*, effective allele; *A1_FREQ*, frequency of the A1 allele; *BETA*, beta coefficients represent the increase in RAG per copy of the A1 allele

In addition, the effects of the 13 SNPs on non-European populations were estimated (Supplementary Table 4). Among these, the effect sizes observed in the Asian population showed the strongest correlation with those in the European population (Pearson correlation coefficient = 0.44, *P* = 0.14), though the correlation was not statistically significant (Supplementary Table 5, Supplementary Fig. 4).

### Functional annotation of the 13 sentinel SNPs identified by MTAG

For each of the sentinel loci, SNPs that were in LD (*r*^2^ ≥ 0.8) were annotated, and all SNPs were mapped to the nearest gene within 10 kb of the SNPs. The majority of the SNPs were intergenic and 28 gene regions were mapped. Two of the sentinel SNPs—rs3851294 on chromosome 1 and rs4143008 on chromosome 8—may have deleterious effects (CADD scores > 12.37); 79% of these SNPs are expression quantitative trait loci (eQTLs) and 2016 chromatin interactions with the 8 leading regions were observed (Supplementary Results 1, Supplementary Tables 6–7 and Supplementary Figs. 5–6).

### In silico* lookup for pleiotropic effects*

**Fig. 4:**
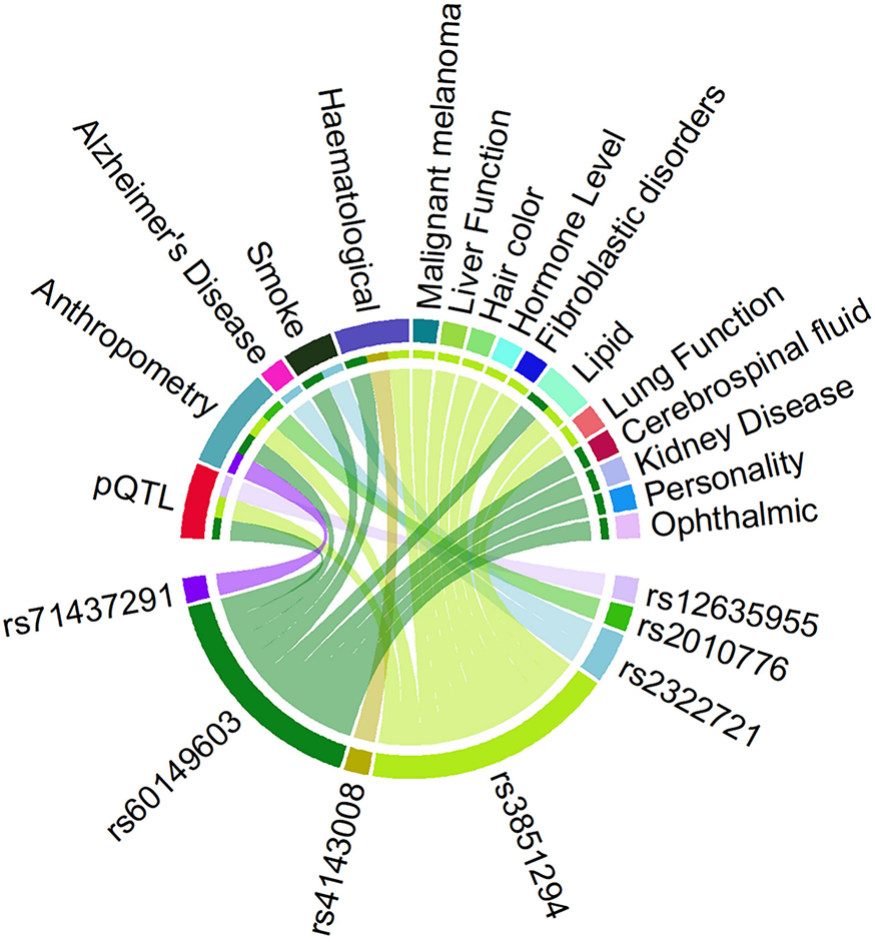
Association of RAG loci with different traits. Plot shows results from associations with other traits which were extracted from the PhenoScanner/GWAS catalog databases for the 14 novel sentinel SNPs including proxies in Linkage Disequilibrium (*r*^2^ ≥ 0.8) with genome-wide significant associations

By looking up the SNPs in PhenoScanner and GWAS catalog, we evaluated the cross-trait and disease associations of the sentinel SNPs identified from MTAG. By selecting traits at a genome-wide significant level (5e − 08), the search of published GWAS showed that 7 of our 13 loci (using sentinel SNPs or proxies estimated in the European population; *r*^2^ ≥ 0.8) were also associated with a wide range of traits and diseases. Among them, anthropomorphic traits such as BMI and body height measures were most commonly shared with RAG SNPs, highlighting a common link between body growth and RAG. Hematological measurements such as mean corpuscular hemoglobin concentration, and white blood cell and basophil counts were also associated with different RAG SNPs, indicating the potential importance of cellular blood constituents in retinal aging. Ophthalmic measurements such as spherical equivalent were also tagged by proxy SNPs. In particular, rs3851294 in *DSTYK* and rs60149603 in *ALKAL2* demonstrated a variety of pleiotropic effects. These findings suggest a complex array of biological processes involved in retinal aging (Fig. 4, Supplementary Table 8).

We further explore the association of sentinel SNPs with lifestyle traits by looking up the PheWAS in the UK Biobank via the Gene ATLAS database (*N* = 408,455). At a moderate level (*P* < 0.05), we found genetic associations of RAG variants with different lifestyle traits. However, the overall associations demonstrated heterogeneous effects with lifestyle. More details are in Supplementary Results 2, Supplementary Table 9, and Supplementary Fig. 7.

### Enrichment analysis

We performed gene-based and pathway-based tests using MAGMA v1.07b in stage 1 and 2 results separately, and lastly in the MTAG result (Supplementary Table 10 and Supplementary Fig. 8a–c demonstrated the prioritized genes of each stage). The genome-wide significance level was defined at *P* = 2.607e − 6 (0.05/19,182). Two genes *SH3YL1* and *ACP1* located on chromosome 2 were prioritized in each of GWAS stages 1, 2, and 3. *SH3YL1* is a newly identified retinal vessel density gene [12], indicating retinal vascular features might also be a feature of RAG. We found two genes—*ARMS2* and *PLEKHA1*—located on chromosome 10 were enriched in GoDARTS data and had *P* values of 7.07e − 04 and 0.03 in MTAG. Though not reaching genome-wide significance, these genes were AMD genes, suggesting that macular degeneration might be a further feature of RAG (Supplementary Fig. 8b). Overall, 9 genes were prioritized for MTAG results; among them, *TMCC2* from chromosome 1 and *CCDC25* from chromosome 2 were newly identified genes (Supplementary Table 10, Supplementary Fig. 8).

None of the enriched pathways was significant after adjustment (FDR < 0.05). Enrichment of the MTAG result suggests a reactome pathway—"melanin biosynthesis”—might be involved in the retinal aging process (*P* = 1.17e − 05). The top pathways are shown in Supplementary Table 11. Both MAGMA and hypergeometric mean pathway analysis (GENE2FUNC implemented in FUMA) did not identify any enrichment of the RAG gene in any tissue. The overall expression pattern of the mapped genes in different tissues and cells is shown in Supplementary Figs. 9 and 10.

### Different biological ages share common genes

**Fig. 5:**
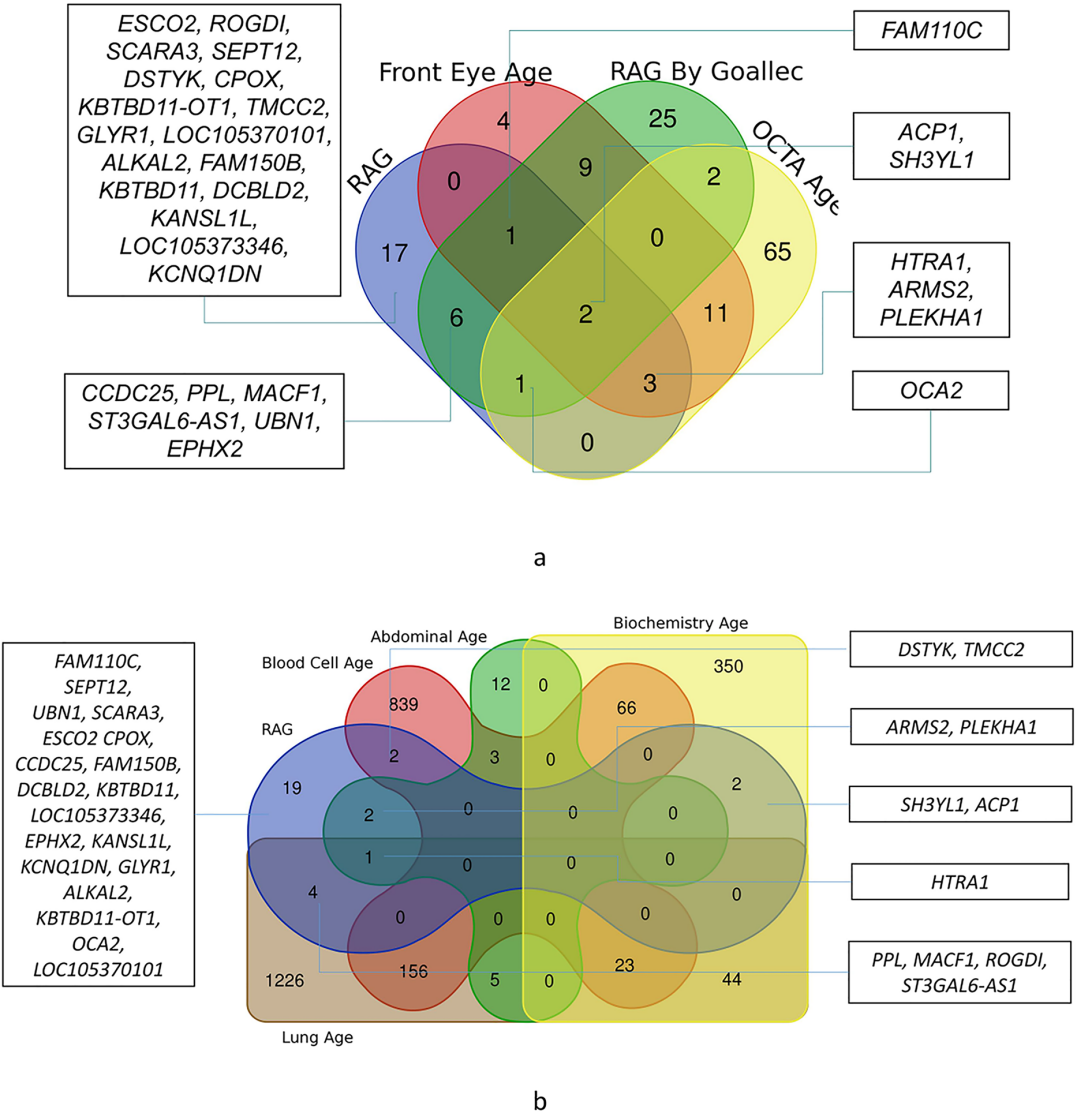
Venn diagram of overlapped genes of different biological ages. Genes for different BAs were reported by Goallec et al. [20], data were derived from https://www.multidimensionality-of-aging.net/. **a** Common genes of RAG between our study and Goallec’s findings. **b** Common genes between RAG and other biological age

For further validation, we compared the genes identified in our study (from stages 1, 2, and 3) with genes of different biological ages identified by Goallec et al. [20]. In Goallec et al.’s study, RAG was calculated based on eye front segment image, fundus image, or OCTA. We note that, among the 30 unique genes reported in our study (from either direct mapping or from enrichment analysis), 13 of them were overlapping with the RAG genes reported by Goallec et al. Especially, 10 genes (*ACP1*, *SH3YL1*, *FAM110C*, *OCA2*, *CCDC25*, *PPL*, *MACF1*, *ST3GAL6-AS1*, *UBN1*, *EPHX2*) were exactly matching with RAG genes derived from retinal fundus images (Fig. 5a). Moreover, 8 of our RAG genes were shared by other biological ages: *HTRA1* was identified as genes for both “abdominal age” and “lungs age,” while *ARMS2* and *PLEKHA1* were genes for “abdominal age” and *PPL*, *ST3GAL6-AS1*, *ROGDI*, and *MACF1* were “lungs age” genes; *SH3YL1* and *ACP1* were identified as genes for “biochemistry age,” and *TMCC2* and *DSTYK* were also a gene for “blood cells age” (Fig. 5b).

### Genetic correlation analysis and effect of genetic risk score

We further applied linkage disequilibrium score regression analysis to test the genetic correlation between RAG and multiple ocular and non-ocular traits. However, none of the results passed the Bonferroni multiple testing. Details are listed in Supplementary Table 12.

The RAG GRS was divided into tertiles. Compared to participants in the lowest tertile, those in the higher tertiles showed an increased risk of being categorized in the accelerated RAG group, with an odds ratio of 1.71 (95%CI 1.48–1.99, *P* < 0.001) for the middle tertile and 3.25 (95%CI 2.80–3.77, *P* < 0.001) for the highest tertile. The RAG GRS was also potentially associated with an increased risk of glaucoma and cataract but not with AMD or all-cause mortality (Supplementary Table 13). Including the RAG GRS in prediction models improved the area under the ROC curve (AUC) for glaucoma and cataract risk prediction (Supplementary Table 14).

### Mendelian randomization analysis: HbA1c and blood lipid, refractive status, and inflammation-related hemocytes might cause retinal aging

We performed a series of two-sample Mendelian randomization studies to assess the putative causal relationships between systemic phenotypes on RAG. To select instrumental variables (IV), for each trait, we removed SNPs that were not genome-wide significant (*P* > 5e − 08), that were in high LD (*r*^2^ > 0.1), and that were not in the MTAG GWAS results. The IVs included are shown in Supplementary Tables 15–18. Although none of the traits passed the threshold of multiple testing correction, several findings were reaching the suggestive significant threshold (*P* < 0.05).

#### Ocular traits

We chose SNPs for spherical equivalent (12 SNPs), axial length (2 SNPs), IOP (51 SNPs), glaucoma (14 SNPs), AMD (5 SNPs), and diabetic retinopathy (2 SNPs) as IV. Interestingly, we found that the smaller spherical equivalent (more myopic) might be causally associated with a greater RAG, although subsequent sensitivity analysis could not support this finding (IVW: beta = − 0.065, *P* = 0.01; MR-Egger beta = 0.13, *P* = 0.33; MR-Egger intercept = − 0.02, *P* = 0.15) (Supplementary Table 15). Besides, IVW analysis also suggested that higher IOP tend to accelerate retinal aging (IVW: beta = 0.022, *P* = 0.04). Although MR analysis did not support the causal effect of AMD on retinal aging, there was a strong heterogeneous effect of the IVs for AMD (*Q* = 15.52, *P* = 0.004), which indicated pleiotropy.

#### Modifiable clinical risks

We found that the increasing genetic predisposition to raised glycated hemoglobin levels (8 SNPs) would cause an increase in RAG (IVW: beta = 0.29, *P* = 0.04; weighted median: beta = 0.34, *P* = 0.01; weighted mode: beta = 0.35, *P* = 0.03; MR-Egger intercept = − 0.004, *P* = 0.52). For the blood lipid level, the increased HDL level seemed not to be associated with accelerated retinal aging (IVW: beta = 0.06, *P* = 0.12), while total cholesterol (IVW: beta = − 0.06, *P* = 0.02), triglycerides (IVW: beta = − 0.10, *P* = 0.03), and LDL level (IVW: beta = − 0.07, *P* = 0.01) seem to be causal factors for delaying the senility retina, which is consistent with previous findings about the effect of blood lipid on AMD [44–47]. Blood pressure, blood urea nitrogen levels, or eGRF levels were not causally associated with retinal aging (Supplementary Tables 16).

#### Hematological measurements

Since in the PheWAS lookup, we found an association between RAG SNPs and hematological measurements, we tested whether there is a causal relationship between hemocyte components and retinal aging. Among the tests, mean corpuscular hemoglobin concentration (IVW: beta = − 0.13, *P* = 0.01; weighted median: beta = − 0.21, *P* = 0.003; weighted mode: beta = − 0.22, *P* = 0.02) and basophil percentage of white cells (IVW: beta = − 0.13, *P* = 0.01) were causing a younger retinal appearance, while the inflammation-related hemocytes, such as neutrophil percentage of white cells (IVW: beta = 0.15, *P* = 0.002), white blood cell count (IVW: beta = 0.10, *P* = 0.01), and granulocyte count (IVW: beta = 0.09, *P* = 0.036), were causing an older retinal appearance (Supplementary Table 17).

#### Anthropometric measures and lifestyle

Finally, we investigate the anthropometric measures and lifestyle traits; no causal relationships were identified by MR (Supplementary Tables 18).

## Discussion

In summary, we used different deep learning algorithms to quantify in vivo retinal age across nearly 40,000 human retinal fundus images from two independent study cohorts. Our study has several main findings: (1) Robustness of DL in predicting RAG: although the defining and the deriving of retinal age by deep learning algorithms have not been fully established, consistent findings from different studies support the robustness of applying deep learning to quantify the retinal age. (2) Biological relevance of RAG: genetic factors are contributing to the accelerated retinal aging, which explains around 15% of the phenotypic variation. (3) Comprehensive causality: acceleration of retinal aging is a consequence of ophthalmic and whole systemic degeneration, including changing of refractive status, blood biochemistry, and hemocyte components, etc. Here, we would like to discuss these findings in more detail.

### Consistent genetic findings for RAG quantified by different DL suggest the robustness of the DL model

To our knowledge, this is the first study that compared the genetic composition of RAG defined by different DL algorithms and we identified highly overlapped genetic etiology of RAG between the UK Biobank study and the GoDARTS study. For these two cohorts, despite their discrepancies in the training population and algorithms, many of the genetic findings were replicated, and the correlation of the SNPs was high (Pearson correlation coefficient = 0.82, Fig. 2). More importantly, the overall genetic correlation between the two RAGs was as high as 0.67 (*P* = 0.021). These findings partially eliminated our concerns, which is when there is no stander or clear definition of what RAG is, will a different model give a totally different trait?

Biological age derived by DL shared common genetic etiologies, suggesting the information extracted by DL, is not artificial. Conventional methods could only extract limited information from these medical data, but DL technology empowers the medical data with more information, such as biological age. As further validation, we compared our findings with Goallec et al.’s [20] findings. In his study, the DL method was applied to different eye images, abdominal MRI, blood test results, and lung functions, and shared genes were identified among different RAGs as well as different biological ages. The concordance of genetic findings between our defined RAG with a third DL-trained RAG provided further evidence of the reproducibility of DL.

### GWAS findings support the biological relevance of RAG

Firstly, we found that RAG calculated by different DL is replicable, which built up the basis for looking for the biological relevance of the trait. Then, by using a hypothesis-free genome-wide association analysis, we explored the genetic factors of RAG. We used MTAG to merge two GWAS results. MTAG is a recently developed meta-analysis method that is known for its robust character against sample overlapping and less concordance in traits [27]. The MTAG boost the power of GWAS and identified new loci for retinal aging. According to our findings, we estimate that genetic factors can explain around 15% of the variation of the trait. In other words, there are genetic factors that determine the “younger” or “order” appearance of the retina compared to the average age-matched population.

The GWASs and gene-based enrichment analyses performed on the RAG traits highlighted genetic variants or genes associated with retinal function or even retinal aging mentioned above by Goallec et al. [20] (Fig. 5a). *SH3YL1* (SH3 And SYLF Domain Containing 1) is the top GWAS signal in our study, and a recent study unveils that *SH3YL1* is a gene regulating the retina vessel density. Together with *OCA2* (oculo-cutaneous albinism 2), which is required for melanosome maturation and involved in post-embryonic morphogenesis in the eye of fish [48], is a newly identified gene for retinal vessel fractal dimension [12]. These findings may suggest that the retinal vessel morphology may alter the blood perfusion hence causing the degeneration. Another set of genes — *PLEKHA1/ARMS2/HTRA1* — located on chromosome 10, were identified in the GWAS for the GoDARTS population only, and are genes for AMD [49–51]. Although not replicated in our GWAS on the UKB population, which is probably due to the relatively “healthier” images being trained in the UKB population, Goallec et al.’s [20], GWAS for RAG on eye images supported our findings. This indicates that AMD genes are driving retinal aging. Among the other replicated RAG genes, microtubule-actin crosslinking factor 1 (*MACF1*) is one of the most abundant proteins of the photoreceptor proteome [52]. This gene is also involved in brain aging [53], brain aging diseases like Parkinson [54], and aging-related bone loss [55]. Another noteworthy gene, *FAM150B/ACP1* (platelet-derived growth factor receptor signalling), is involved in myopia development [56]. In addition, *PPL* and *ST3GAL6-AS1* were found for accelerating the aging process of the lungs or abdominal (Fig. 5b), while *CCDC25* is related to Alzheimer’s disease [57]. The partially overlapping aging genes across different organs suggest that while chronological age is uniform, the biological age may vary across different tissues and organs.

Several newly identified RAG genes also demonstrated effects on age-related phenotypes: *TMCC2* binds with APOE [58], which is a well-known gene for longevity; *DSTYK* is involved in the skin aging process [59]; *SCARA3* is highly expressed in autosomal recessive retinitis pigmentosa eyes [60]; *KCNQ1DN* is hypermethylated upon aging and was used as a biomarker to predict epigenetic aging [61].

In summary, by performing a GWAS on RAG, we identified the potential genetic contributions to accelerated retinal aging which support its biological relevance. This further supports that RAG might be a good biomarker for aging as it also reflects the systemic aging process.

### Both ophthalmic and systemic factors accelerate retinal aging

Identifying the causal factors for RAG is important. As a newly emerged concept, “accelerated retinal age” is gradually gaining attention, but this artificially defined trait remains the “black box” nature inherited from DL. On one hand, exploring its potential in the future disease prediction area could maximize its clinical application; while on the other hand, investigating its biological relevance or even its biological causal mechanisms could help demystify the “black box”and reveal the etiologies driving accelerated retinal degeneration. By using the technique of Mendelian randomization, we assess the biological causal factors for RAG from the genetic basis for the first time. In our MR study, although none of the traits passed Bonferroni correction, we still value the findings suggested by MR. In general, the 95% CIs of the IV estimates were wider compared to those of the conventional regression, and hence a significant finding via IV analysis (*P* < 0.05) usually indicating strong phenotype associations. Besides, we applied a series of sensitivity analyses for each trait to increase the robustness of our findings [62, 63].

According to our MR findings, we notice that the IVW method suggests that myopia might accelerate retinal aging. This is contradictory to the observational study that a positivist association was observed between SE and chronological age. This might be due to the following reasons: (1) RAG reflects the biological function of the retina in a better way compared to the chronological age. Myopia, especially high myopia, would cause thinning of the retinal neuron layer and increase the risk of retinal detachment and AMD [64], which are all causes of retinal degeneration and vision loss. (2) RAG represent the retinal status other than the overall ocular refractive situation. The positivist association between SE and chronological age is caused by presbyopia—a consequence of the loss of the ability for the eye to accommodate to focus on nearby objects. This is mainly due to the loss function of the lens but not the retina, RAG might not capture this information.

For the modifiable clinical risk factors, we notice that HbA1c is a strong causal factor for RAG. Elevated HbA1c is the consequence of long-term exposure to hyperglycemia, and is the hallmark of T2D. Glycosylation and reactive oxygen are induced during the hyperglycemia exposure, which would cause vascular endothelial dysfunction or death, leading to a leakage of the microvessels [65, 66]. The clinical manifestations are featured as micro-aneurysm, exudes, or even neovascularization [67], which influence the function of the retina and might be the marker of retinal aging. Besides, our MR results suggest elevated LDL and cholesterol are associated with a younger appearance of the retina. Different from cardiovascular disease, many MR studies also reported a risk role of HDL and protective roles of LDL/cholesterol/TG on AMD [44, 68, 69]. These findings from epidemiologic studies may neglect the competing risk of death [70]. By far, no clear mechanism can explain this phenomenon. According to previous studies, during the formation of HDL and the lipid transportation process within the retina, lipoprotein was accumulated and acts as a barrier for lipid transport through an aging retina, leading to the formation of drusen, the marker of AMD [46]. Meanwhile, HDL may contain essential complement components which involve the major pathway in AMD pathogenesis [71]. Our findings highlight the value of studying the biological age of an organ, since different organs are susceptible to different risk factors.

We also notice that increased hemoglobin concentration is causally associated with a younger appearance of the retina while increased white cell counts and neutrophil percentage accelerate retinal aging. There is rich blood perfusion into the retinal choroid and the retinal neuron layer is nourished by the ocular vessel system. Anemia is featured as low hemoglobin concentration and leads to low oxygen-carrying capacity in the blood. Anemia causes retinal hypoxia, which can lead to infarction of the nerve fiber layer and clinically manifests as cotton wool spots. Retinopathy is a frequent finding in anemic and thrombocytopenic patients and our findings confirmed this finding [72]. White blood cells and neutrophils are the inflammatory markers. During the inflammation reaction process, reactive oxygen species are generated, while the oxidative stress is associated with a wide range of retinal diseases such as AMD [73], diabetic retinopathy [74], and some rare retinal diseases such as Leber hereditary optic neuropathy [75]. For systemic diseases, with the aging process, granulocyte counts were increased which is also related to Parkinson’s disease [76]. These findings indicate that the hemocyte components are also related to retinal aging and RAG also unveils the systemic aging process.

## Limitations

The comparability of different DL methods needs to be assessed systematically by comparing each other within the same population. During the analysis, we found that both the UKB model and the GoDARTS model tend to shrink the distribution of the predicted retinal age, e.g., for people with a younger chronological age, their predicate age tends to be older and vice versa. To overcome this problem, in our GWAS analysis, we used the rank transformation to overcome this problem partially. Lastly, our study is unable to describe the environmental effect of retinal aging; hence, epidemiology studies are needed.

## Conclusion

In summary, we discovered and replicated the genomic determinants of retinal biological age estimated by DL applied to fundus images by GWAS. We also explored the potential causalities through a series of MR analyses. Our findings have several contributions: (1) the concordance findings between two independent cohorts reassumed robustness of DL in predicting retinal imaging-based ages; (2) GWA results and the following bioinformatic analyses provided the genetic etiology of retinal aging; (3) MR findings indicated that the retina is particularly vulnerable to risk factors such as elevated glycated hemoglobin, inflammation factors, or anemia; hence, accelerated retinal age might reflect the corresponding systemic conditions. Our findings expanded the current knowledge of the biological implications of retinal aging as well as provided potential therapeutic targets for the general aging process.

## Supplementary Information

Below is the link to the electronic supplementary material.

Supplementary file1 (DOCX 3.84 MB)

Supplementary file2 (XLSX 205 KB)

Supplementary file3 (DOCX 20.8 KB)

## Data Availability

Data are available in a public, open-access repository (https://www.ukbiobank.ac.uk). The GWAS summary statistics used in this study for genetic correlation or MRanalysis are available via GWAS Catalog, under study accession identifiers GCST007461, GCST006368, GCST001032, GCST002647, GCST007982, GCST007327, GCST008062, GCST002221, GCST006630, GCST008059, GCST007954, GCST002223, GCST002222, GCST006629, GCST006624, GCST002216, GCST001884, GCST002115, GCST006065, GCST005580, GCST006424, GCST006289, GCST005195, GCST006906, GCST007511, GCST006867, GCST006414, GCST008064, GCST008598, GCST009890, GCST009413, GCST90011766, GCST009404, GCST009411, GCST009412, GCST009414, or PMID:29,227,965. Summary statistics of body height and BMI were acquired from GIANT consortium (https://portals.broadinstitute.org/collaboration/giant/index.php/GIANT_consortium_data_files). The data and code used in this current study are available from the corresponding authors on a reasonable request.
